# Study of tumor budding and its association with clinicopathological parameters in breast carcinoma

**DOI:** 10.1590/1806-9282.20240173

**Published:** 2024-08-16

**Authors:** Suresh Kaundiyal, Smita Chandra, Anshika Arora

**Affiliations:** 1 Swami Rama Himalayan University, Himalayan Institute of Medical Sciences, Department of Pathology – Dehradun, India.; 2 Swami Rama Himalayan University, Himalayan Institute of Medical Sciences, Department of Surgery – Dehradun, India.

**Keywords:** Breast neoplasms, Tumor budding, Tumor, Buds

## Abstract

**OBJECTIVE:**

Tumor budding is a phenomenon in which the tumor cells detach from the main mass and are present at the invasive front. The present study was conducted to study tumor budding in invasive breast carcinoma and to correlate it with clinicopathological parameters and molecular subtypes.

**METHODS:**

The study was conducted over a period of 1 year, and tumor budding was studied as a single or group of cells at the invasive front of breast carcinoma counted in a high-power field (40×). The grading was statistically correlated with tumor size, grade, lymph node status, lymphovascular invasion, pathological TNM staging, molecular subtype, and survival of patients.

**RESULTS:**

A total of 50 cases of invasive breast carcinoma were included, out of which 66% (n=33) showed high-grade tumor budding, which was statistically significantly higher in grade 2 invasive ductal carcinoma (p<0.05). High tumor budding was associated with lymphovascular invasion, lymph node metastasis, and a high Ki-67 proliferative index. All cases showing low-grade budding were alive until 6 months of diagnosis, but there was no statistically significant association between stage and budding.

**CONCLUSION:**

Tumor buds are significantly higher in grade 2 invasive ductal carcinoma with lymphovascular invasion, lymph node metastasis, and a high Ki-67 proliferative index. Immunohistochemistry may prove helpful in distinguishing tumor buds from their mimickers. Further studies with extended follow-up are recommended to predict tumor budding as a prognostic marker in breast carcinoma, which may play an important role in cancer therapy.

## INTRODUCTION

Breast cancer is the most common cancer in the world, with an age-standardized incidence rate of 47.8 and a mortality rate of 13.6 per 100,000 population^1^. It has been estimated that slightly more cases of breast cancer are present in less developed areas of the world than in more developed areas. Tumor budding is a phenomenon in which the tumor cells become detached from the main tumor mass and are present at the invasive front^2^. It has been considered to play an important role as a prognostic factor^3^. Tumor budding has been studied in different carcinomas, and the International Tumor Budding Consensus Conference (ITBCC) has highlighted a scoring system for the reporting of tumor budding in colorectal cancer^4^. The present study was therefore conducted to study tumor budding in invasive breast carcinoma and to correlate it with clinicopathological parameters and molecular subtypes.

## METHODS

**Figure 1:**
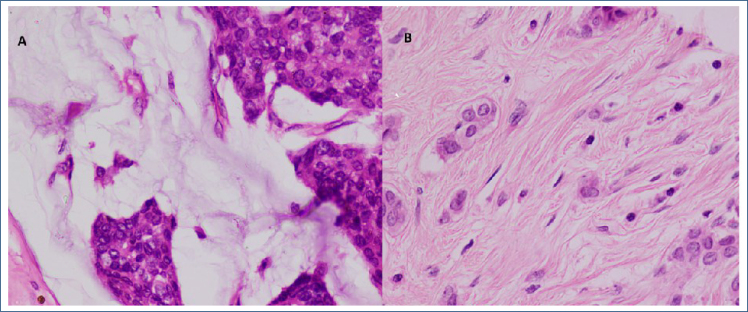
(A) Section shows invasive ductal carcinoma having low-grade tumor budding and (B) section shows invasive ductal carcinoma having high-grade tumor budding (H&E, 40×).

**Figure 2:**
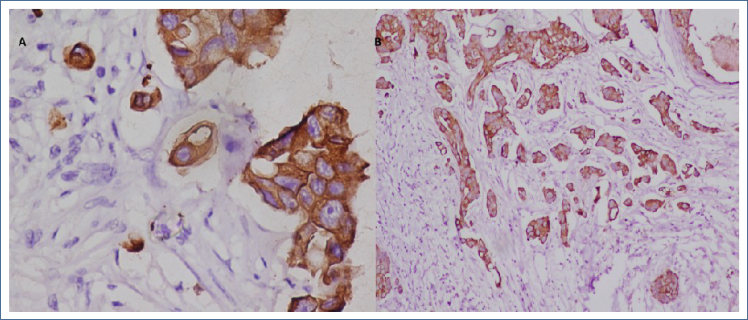
(A) A pan-CK-stained section demonstrates low-grade tumor buds in invasive ductal carcinoma and (B) a pan-CK-stained section demonstrates high-grade tumor buds in invasive ductal carcinoma (immunohistochemical pan-CK stain, 40×).

The study was conducted in the Department of Pathology over a period of 1 year and included all the newly diagnosed cases of invasive breast carcinoma. The core biopsies were excluded from the study. Relevant clinical details were noted for every case, and hematoxylin and eosin-stained sections were studied for histomorphological features, grading, and TNM staging according to the WHO classification of breast tumors^5^. Tumor budding was studied in every case as per the recommendations of the ITBCC, 2017^6^. Either a single or a group of five cells at the invasive front of breast carcinoma were counted in a high-power field (40×) as tumor buds (Figure 1). These tumor buds were counted in 10 high-power fields and documented as low- or high-grade depending on the number of buds. High-grade tumor budding was considered when tumor buds were >20/10 HPF and low when tumor buds were ≤20/10 HPF. The immunohistochemical examination was done for every case for ER, PR, HER2 neu, and Ki-67 to determine the molecular subtype of breast carcinoma. Pan-CK immunohistochemical staining was also done for confirmation of tumor buds (Figure 2). The tumor buds grading was then statistically correlated with clinical features and histopathological parameters, including tumor size, grade, lymph node status, lymphovascular invasion, pathological TNM staging, molecular subtype, and survival of breast carcinoma patients.

Statistical analysis of the observations was performed using the SPSS software (Statistical Package for Social Sciences) version 23 and Microsoft Excel. Categorical data was expressed as frequencies, and continuous data as mean±standard deviation or median. The association of categorical variables was analyzed using Pearson's chi-square test. A p-value of <0.05 was considered significant. The study was approved by the institutional research and ethics committee via letter no. SRHU/HIMS/RC/2022/108 dated April 2, 2022.

## RESULTS

The study included 50 cases of invasive breast carcinoma, with all the cases being female. The mean age was 48.66±12.25 years, the median was 47 years, and the age range was 25–79 years. The right breast was involved in 56% of cases (n=28), while 2% (n=1) of cases showed bilateral breast involvement, with upper quadrant involvement in 62% of cases (n=31). Most commonly, the cases (n=43, 86%) presented with a lump in the breast for a duration of more than 6 months and bloody nipple discharge in 2% of cases. On mammography, 58% of cases (n=29) were in the BIRADS 4c (Breast Imaging Reporting and Data System) category, and on FNAC, 98% of cases were diagnosed as ductal carcinoma. On gross examination of the mastectomy specimen, a tumor size of 2–5 cm was observed in 60% of cases (n=30), with ulcero-infiltrative growth in 98% of cases. Histologically, 47 cases were of invasive ductal carcinoma, 2 were of invasive lobular carcinoma, and 1 was of mucinous carcinoma. Table 1 shows the distribution of histopathological findings in the carcinoma cases. It shows that grade 2 (RB score of 6–7) was present in 72% (n=36) of cases. The maximum number of cases, 22% (n=11), were pT2N0Mx, followed by pT2N1aMx at 16% (n=8), and 96% (n=48) of cases were alive after 6 months of diagnosis. The immunohistochemical staining for ER, PR, HER2 neu, and Ki-67 revealed that 48% (n=24) were luminal B breast carcinoma, while 24% were triple-negative breast carcinoma. It was observed that 66% of cases (n=33) showed high-grade tumor budding, which was statistically significantly higher in invasive ductal carcinoma, grade 2 (p<0.05). It was also observed that high tumor budding was associated with grade 2 breast carcinoma and lymphovascular invasion, and 77.14% (n=27) of cases showing high-grade tumor budding had lymph node involvement by the carcinoma. It was also observed that 91.4% of cases with a high Ki-67 proliferative index showed high-grade tumor budding. Table 2 shows the association between tumor budding and the molecular classification of breast carcinoma. It shows that triple negative and luminal B type of breast cancer had low-grade tumor budding in 35.3% (n=6) of cases, and luminal B type had high-grade tumor budding in 54.5% (n=18) of cases. Although it was observed that 100% of cases showing low-grade tumor budding were alive until 6 months of diagnosis, there was no statistically significant association between stage and tumor budding.

**Table 1:** Distribution of breast carcinoma cases according to the histopathological findings.

Histopathological findings		Number of cases (n)	Percentage (%)
RB score	Grade I (score 3–5)	4	8
	Grade II (score 6–7)	36	72
	Grade III (score 8–9)	10	20
Intratumoral DCIS (>25%)		8	16
Intratumoral DCIS (<25%)		42	84
Extratumoral DCIS (>10%)		4	8
Necrosis	Not seen	0	0
	Focal	18	36
	Extensive	32	64
Calcification		3	6
Lymphovascular invasion		39	78
Perineural invasion		4	8
Uninvolved breast	Fibrocystic breast disease	44	88
	Chronic mastitis	6	12

TNM: tumor node metastasis; FNAC: fine-needle aspiration cytology; DCIS: ductal carcinoma in situ.

**Table 2:** Association between tumor budding and molecular classification of breast carcinoma.

Molecular classification	Tumor budding			p-value
	Low grade	High grade	Total	
Luminal A	0 (0.0%)	3 (9.1%)	3 (6.0%)	0.219
Luminal B	6 (35.3%)	18 (54.5%)	24 (48.0%)	
HER2 neu positive	5 (29.4%)	6 (18.2%)	11 (22.0%)	
Triple negative	6 (35.3%)	6 (18.2%)	12 (24.0%)	
Total	17 (100.0%)	33 (100.0%)	50 (100.0%)	

## DISCUSSION

Tumor budding, which is the phenomenon of the separation of a cluster of tumor cells from the main tumor mass, is considered the initial stage of metastasis^7^. It has been studied in various carcinomas, including lung carcinoma and head and neck carcinoma, and is considered to have prognostic significance^8,9^. Although the present study observed tumor budding in different histomorphological types of breast carcinoma, including ductal, lobular, and mucinous carcinoma, most of the previous studies have studied it in only invasive ductal carcinoma^10,11^. The observation of tumor buds may be done with 40× or 20× objective lens, but it is considered that at low power, it may become difficult to differentiate tumor buds from other cells^2,10,12^. The present study observed tumor buds at 40× and also confirmed them by doing immunohistochemical staining with cytokeratin. Liang et al. also confirmed tumor buds by doing immunohistochemical staining, which excluded any mimickers^13^. The authors therefore suggest that immunohistochemical cytokeratin stain may be helpful if there is any confusion regarding the presence of tumor buds, but in routine practice, observation at 40× may be sufficient.

An important finding observed in the present study was that high tumor budding was associated with grade 2 breast carcinoma, lymphovascular invasion, lymph node metastasis, and a high Ki-67 proliferative index. Previous studies have also observed similar findings, but the correlation with a high proliferative index is limited^14,15^. This suggests that tumor budding may emerge as an important prognostic factor in breast carcinoma. However, Mozarowski et al. observed in their study that there is no statistically significant difference in the frequency of complete or partial responses between the group having tumor budding and another without it^16^. In contrast, Silva et al. concluded that tumor budding in early breast cancer is a novel factor in the determination of adjuvant therapy decisions by identifying patients at a high risk of relapse and benefiting from treatment intensification^17^.

It was observed in the present study that all the patients with low tumor budding survived for at least 6 months. Although this may suggest that tumor budding may be associated with survival, the follow-up period is too short for a definite opinion about it. Okcu et al. recently concluded that tumor budding is a reliable predictor of death and metastasis in invasive ductal breast cancer^18^. It has also been observed previously that a sentinel lymph node biopsy showing extracapsular extension was associated with additional positive axillary lymph nodes^19^.

It has been reported that tumor budding is associated with epithelial-mesenchymal transition and interacts with the tumor microenvironment for metastasis^20^. Recently, partial epithelial-mesenchymal transition, which is a hybrid state in which both epithelial and mesenchymal characteristics are studied in relation to tumor budding, may be helpful in adjuvant therapy planning^20–22^. Previously, it has been observed that breast-conserving surgery is adequate for overall survival than mastectomy, even in large lesions, and is associated with a higher pathological complete response^23^. The combination of estrogen with melatonin has also been studied for breast cancer survivors, especially in females with intense vasomotor symptoms, and further studies are recommended for optimal hormonal replacement^24^.

An important limitation of the present study was that only a limited number of cases were studied, with a survival period of only 6 months, which may not be enough to sufficiently comment on tumor budding as a prognostic marker in breast carcinoma.

Thus, to conclude, tumor buds are significantly higher in grade 2 invasive ductal carcinoma and are associated with lymphovascular invasion, lymph node metastasis, and a high Ki-67 proliferative index. It has to be histomorphologically studied at 40× to differentiate from other mimicking cells. Although immunohistochemistry using the epithelial marker pan-CK may prove helpful if there is any difficulty in differentiation of malignant cells from inflammatory cells, mostly routine HE-stained sections are sufficient. Further studies with extended follow-up are recommended to predict tumor budding as a prognostic marker in breast carcinoma and thus may play an important role in cancer therapy.

## References

[1] Ferlay J, Ervik M, Lam F, Colombet M, Mery L, Piñeros M (2020). Global cancer observatory: cancer today.

[2] Kumarguru BN, Ramaswamy AS, Shaik S, Karri A, Srinivas VS, Prashant BM (2020). Tumor budding in invasive breast cancer - An indispensable budding touchstone. Indian J Pathol Microbiol.

[3] Lugli A, Zlobec I, Berger MD, Kirsch R, Nagtegaal ID (2021). Tumour budding in solid cancers. Nat Rev Clin Oncol.

[4] Zlobec I, Berger MD, Lugli A (2020). Tumour budding and its clinical implications in gastrointestinal cancers. Br J Cancer.

[5] Lakhani SR, Ellis IO, Schnitt SJ, Tan PH, Vijver MJ (2012). WHO classification of tumours of the breast.

[6] Lugli A, Kirsch R, Ajioka Y, Bosman F, Cathomas G, Dawson H (2017). Recommendations for reporting tumor budding in colorectal cancer based on the International Tumor Budding Consensus Conference (ITBCC) 2016. Mod Pathol.

[7] Voutsadakis IA (2018). Prognostic role of tumor budding in breast cancer. World J Exp Med.

[8] Kadota K, Yeh YC, Villena-Vargas J, Cherkassky L, Drill EN, Sima CS (2015). Tumor budding correlates with the protumor immune microenvironment and is an independent prognostic factor for recurrence of stage I lung adenocarcinoma. Chest.

[9] Shimizu S, Miyazaki A, Sonoda T, Koike K, Ogi K, Kobayashi JI (2018). Tumor budding is an independent prognostic marker in early stage oral squamous cell carcinoma: with special reference to the mode of invasion and worst pattern of invasion. PLoS One.

[10] Sriwidyani NP, Alith-Artha G, Mantik-Astawa N, Manuaba IBTW (2016). Tumor budding in breast carcinoma: relation to E-Cadherin, MMP-9 expression, and metastatic risk. Bali Med J.

[11] Salhia B, Trippel M, Pfaltz K, Cihoric N, Grogg A, Lädrach C (2015). High tumor budding stratifies breast cancer with metastatic properties. Breast Cancer Res Treat.

[12] Gujam FJ, McMillan DC, Mohammed ZM, Edwards J, Going JJ (2015). The relationship between tumour budding, the tumour microenvironment and survival in patients with invasive ductal breast cancer. Br J Cancer.

[13] Liang F, Cao W, Wang Y, Li L, Zhang G, Wang Z (2013). The prognostic value of tumor budding in invasive breast cancer. Pathol Res Pract.

[14] Agarwal R, Khurana N, Singh T, Agarwal PN (2019). Tumor budding in infiltrating breast carcinoma: correlation with known clinicopathological parameters and hormone receptor status. Indian J Pathol Microbiol.

[15] Rathod GB, Desai KN, Shrivastava A, Maru AM (2022). Correlation of tumor budding with known clinicopathological, histomorphological and hormonal receptor status in patients with invasive breast carcinoma. Cureus.

[16] Mozarowski P, Rasaiah B, Reed M, Lewis A, Walde N, Voutsadakis IA (2021). Prognostic role of tumor budding in breast cancer patients receiving neo-adjuvant therapy. J Clin Med.

[17] Silva DJ, Miranda G, Amaro T, Salgado M, Mesquita A (2023). Prognostic value of tumor budding for early breast cancer. Biomedicines.

[18] Okcu O, Öztürk Ç, Şen B, Arpa M, Bedir R (2021). Tumor Budding is a reliable predictor for death and metastasis in invasive ductal breast cancer and correlates with other prognostic clinicopathological parameters. Ann Diagn Pathol.

[19] Freitas GB, Mota BS, Maesaka JY, Pinheiro CC, Lima LGCA, Soares JM (2023). Measurement of extracapsular extension in sentinel lymph node as a possible predictor of residual axillary disease in breast cancer. Clinics (Sao Paulo).

[20] Ling Z, Cheng B, Tao X (2021). Epithelial-to-mesenchymal transition in oral squamous cell carcinoma: challenges and opportunities. Int J Cancer.

[21] Argon A, Öz Ö, Kebat TA (2023). Evaluation and prognostic significance of tumor budding in pancreatic ductal adenocarcinomas. Indian J Pathol Microbiol.

[22] Okuyama K, Suzuki K, Yanamoto S (2023). Relationship between tumor budding and partial epithelial-mesenchymal transition in head and neck cancer. Cancers (Basel).

[23] Nobrega GB, Mota BS, Freitas GB, Maesaka JY, Mota RMS, Goncalves R (2023). Locally advanced breast cancer: breast-conserving surgery and other factors linked to overall survival after neoadjuvant treatment. Front Oncol.

[24] Soares JM, Mota BS, Nobrega GB, Filassi JR, Sorpreso ICE, Baracat EC (2023). Breast cancer survivals and hormone therapy: estrogen and melatonin. Rev Assoc Med Bras (1992).

